# Emergence of a high-risk multidrug-resistant *Acinetobacter baumannii* clone ST697 in nosocomial settings

**DOI:** 10.1128/spectrum.02293-25

**Published:** 2026-04-16

**Authors:** Jing Guan, Huiqi Qu, Lin Yu, Weilong Li, Garnet Eister, Hakon Hakonarson, Shaoqiang Li

**Affiliations:** 1 State Key Laboratory of Respiratory Disease, Department of respiratory, National Clinical Research Center for Respiratory Disease, National Center for Respiratory Medicine, Guangzhou Institute of Respiratory Health, the First Affiliated Hospital of Guangzhou Medical University 664066, Guangzhou, People's Republic of China; 2 Department of Laboratory Medicine, The First Affiliated Hospital of Guangzhou Medical University 117969, Guangzhou, Guangdong, China; 3 Center for Applied Genomics, Children’s Hospital of Philadelphia, Philadelphia, Pennsylvania, USA; 4 Department of Laboratory, The Key Laboratory of Advanced Interdisciplinary Studies, The First Affiliated Hospital of Guangzhou Medical University, KingMed School of Laboratory Medicine, Guangzhou Medical University 26468, Guangzhou, China; 5 Department of Pediatrics, The Perelman School of Medicine, University of Pennsylvania 6572, Philadelphia, Pennsylvania, USA; 6 Division of Human Genetics, Children’s Hospital of Philadelphia 24178 , Philadelphia, Pennsylvania, USA; 7 Division of Pulmonary Medicine, Children’s Hospital of Philadelphia 24178 , Philadelphia, Pennsylvania, USA; 8 Faculty of Medicine, University of Iceland 63541, Reykjavík, Iceland; University of Minnesota Twin Cities, Minneapolis, Minnesota, USA

**Keywords:** *Acinetobacter baumannii*, ST697, multi-drug resistance, combined drug susceptibility test, antibiotic combination therapy

## Abstract

*Acinetobacter baumannii* is a critical opportunistic pathogen increasingly linked to hospital-acquired infections due to its ability to acquire multidrug resistance (MDR). Its persistence in clinical environments and resistance to nearly all major antibiotic classes make it a formidable challenge for infection control and treatment. This study investigated the molecular epidemiology and therapeutic strategies for *A. baumannii* across three hospital campuses. A total of 148 clinical MDR *A. baumannii* isolates were collected, mostly recovered from respiratory tract specimens in ICU settings. All isolates underwent whole-genome sequencing (WGS), antibiotic susceptibility testing against 16 antibiotics, and *in vitro* evaluation of 14 antibiotic combinations. WGS confirmed 140 clinical isolates as *A. baumannii,* and 42.3% were classified as extensively drug-resistant (XDR). High resistance rates were observed for carbapenems (98.6%), fluoroquinolones (100%), and aminoglycosides (91.4%), while susceptibility to polymyxin B (PMB) and tigecycline (TGC) remained largely preserved. Four antibiotic combinations, TGC + cefoperazone/sulbactam (CSL), TGC + PMB, PMB + CSL, and minocycline (MNO) + CSL, demonstrated strong synergistic effects (>90%). The efficacy and safety of the MNO + CSL combination were preliminarily assessed in the mouse model. Genome sequence typing revealed ST2 as the dominant clone (92.9%), while a novel XDR clone, ST697, was identified. Genomic and virulence profiling suggests that ST697 evolved from ST2, representing a high-risk lineage with potential for early, rapid dissemination. A notably high mortality rate (71.4%) was observed among patients infected with ST697. Long-read sequencing assembled complete ST697 genomes revealed that ST697 isolates carry multiple copies of the *bla*_OXA-23_ gene, and the *Galleria mellonella* infection model revealed that ST697 isolates exhibit virulence comparable to the highly virulent ST2 *A. baumannii*.

**IMPORTANCE:**

This study reveals the emergence of ST697, a novel, highly drug-resistant, and virulent clone of *Acinetobacter baumannii*, closely related to ST2. It also identifies potent antibiotic combinations that may serve as effective alternatives to last-line monotherapy. These findings highlight the urgent need for surveillance, infection control, and targeted therapy, especially in ICU and respiratory settings, to curb the spread of this emerging clone.

## INTRODUCTION

*Acinetobacter baumannii* is an extremely prevalent gram-negative pathogen responsible for a wide range of infections, including respiratory tract infections, bloodstream infections, urinary tract infections, and central nervous system infections (1). Presently, *A. baumannii* poses a significant global threat to human health and has been designated a critical-priority pathogen by the World Health Organization due to its resistance profile and limited treatment options. The mortality rate associated with *A. baumannii* infection (ABI) ranges from 10% to 60% (2–4) across different populations and may be even higher in cases of severe infection or hospital-acquired pneumonia. The transmission modes of *A. baumannii* are diverse, including direct contact with contaminated medical devices, healthcare workers, and patients, as well as via airborne particles and water sources (5). *A. baumannii* has undergone selective adaptation within hospital environments, resulting in high tolerance to harsh conditions such as disinfectants, desiccation, and oxidative stress (6). This resilience enables the pathogen to persist and spread rapidly in clinical settings. As an opportunistic pathogen, ABI is frequently observed in healthcare facilities, particularly in intensive care units (ICUs) (7–10), due to the high-risk nature of ICU environments, frequent mechanical ventilation, prolonged antibiotic exposure, and compromised immune status of patients.

In recent years, the widespread use of antibiotics, especially carbapenems, has accelerated resistance in *A. baumannii* (11), leading to the emergence of multidrug-resistant (MDR) and extensively drug-resistant (XDR) strains. Most clinical isolates now exhibit resistance to multiple antibiotic classes, including β-lactams, aminoglycosides, and fluoroquinolones (7, 12). This resistance is largely mediated by the acquisition of diverse antibiotic resistance genes (ARGs), such as the class D β-lactamase genes *bla*_OXA23_ and *bla*_OXA-51_, often carried on mobile genetic elements such as plasmids and transposons, facilitating rapid horizontal transfer among bacterial populations (13). The dissemination of MDR and XDR *A. baumannii* (MDRAB and XDRAB) in healthcare environments not only increases infection risk but also complicates treatment and elevates mortality.

Due to the frequent occurrence of MDRAB, therapeutic options are severely limited. Although current resistance to last-line antibiotic therapy such as tigecycline and polymyxin remains relatively low, resistance is expected to rise with increased use. Alarmingly, cases of polymyxin-resistant or tigecycline-resistant *A. baumannii* are no longer rare (14–17). At present, combination antibiotic therapy is the primary treatment strategy for MDRAB and XDRAB, involving the concurrent use of two or more antibiotics to enhance efficacy and delay resistance development. However, treatment success depends on identifying synergistic combinations through laboratory-based susceptibility testing (18–20). *In vitro* studies have shown that synergistic or additive combinations can significantly reduce mortality and extend the clinical utility of existing antimicrobials (21). In this regard, accurate antimicrobial susceptibility testing (AST) plays a critical role in guiding effective therapy and slowing resistance emergence (22, 23).

Molecular typing techniques, including multi-locus sequence typing (MLST) and whole-genome sequencing (WGS), have been extensively used in epidemiological studies of *A. baumannii* (24–27). These tools provide insights into clonal relationships, resistance evolution, and transmission pathways. Among the known sequence types, ST2, also referred to as global clone 2, is the most prevalent lineage globally. In China, ST2 *A. baumannii* has been reported to account for over 80% of clinical isolates from multiple institutions (7, 10). The ST2 lineage is particularly notorious for harboring a broad array of ARGs, contributing to its high levels of multidrug resistance. In addition to resistance, *A. baumannii*’s ability to form biofilms contributes significantly to persistent colonization of the respiratory tract, especially in mechanically ventilated patients (28), and enhances resistance to both antimicrobials and host immune responses and further complicates eradication efforts.

This study aimed to monitor the molecular epidemiological trends of nosocomial MDRAB in a large general hospital, evaluate the effectiveness of antibiotic combination therapies, and identify promising treatment options. Whole-genome sequencing was performed on 148 clinical isolates collected from various departments across three hospital campuses over a 2-year period. A comprehensive investigation was conducted, encompassing sequence typing, evolutionary relationships, ARGs, virulence factor genes (VFGs), antimicrobial susceptibility profiles, and virulence phenotypes. A novel, highly drug-resistant, and virulent *A. baumannii* clone ST697, closely related to the globally predominant ST2 lineage, was identified, with substantial potential for early, rapid dissemination in clinical settings. Additionally, 14 antibiotic combinations were tested to explore novel therapeutic strategies, and 4 were found to exhibit efficacy against MDRAB and XDRAB. Among these four candidates, one was further validated to be effective in the mouse model. These findings provide new insights for optimizing the treatment of MDRAB and underscore the urgent need for enhanced surveillance, strict infection control measures, and targeted antimicrobial therapy, especially in ICU and respiratory care settings, to mitigate the spread of this emerging high-risk clone.

## MATERIALS AND METHODS

### Clinical isolates and public data collection

Clinical isolates were collected from the microbiology laboratories of three campuses of the First Affiliated Hospital of Guangzhou Medical University between January 2021 and October 2022. Species identification was initially performed using the VITEK-MS automated microbial identification system (bioMérieux, Marcy l’Etoile, France). Isolates identified as *A. baumannii* and exhibiting MDR or XDR phenotypes were selected for further analysis. To avoid duplication, only the first isolate per patient meeting inclusion criteria was retained. Basic clinical information, including patient age, gender, length of hospital stay, infection-related markers, and other relevant data, was collected. Infections were classified as hospital-acquired or community-acquired based on whether symptoms developed more than 48 hours after admission. Public *A. baumannii* genomes were retrieved from the National Center for Biotechnology Information (NCBI) genome database. Metadata, including geographic location, release date, and submitter information, was obtained from the NCBI BioSample database.

### Antimicrobial susceptibility testing

AST was performed on 16 antibiotics: amikacin (AMK), cefepime (FEP), cefoperazone/sulbactam (CSL), ceftazidime (CAZ), ciprofloxacin (CIP), polymyxin B (PMB), doxycycline (DOX), imipenem (IPM), levofloxacin (LEV), meropenem (MEM), minocycline (MNO), piperacillin/tazobactam (TZP), sulfamethoxazole (SXT), ticarcillin/clavulanate (TIC), tigecycline (TGC), and tobramycin (TOB). Testing was conducted using the VITEK 2 Compact system (bioMérieux, Marcy l’Etoile, France), with *Escherichia coli* ATCC 25922 and *Pseudomonas aeruginosa* ATCC 27853 as quality control strains. Antibiotic breakpoints were interpreted based on the Clinical and Laboratory Standards Institute 2022 guidelines. For TGC, US Food and Drug Administration criteria specific to *Acinetobacter* spp. were applied: MIC ≤2 mg/L (susceptible), 4 mg/L (intermediate), and ≥8 mg/L (resistant); disk diffusion ≥16 mm (susceptible), 13 mm–15 mm (intermediate), and ≤12 mm (resistant). Isolates with intermediate or resistant tigecycline results were confirmed using the broth microdilution method. Isolates resistant to at least two antimicrobial classes were classified as MDR, while those resistant to all tested agents except PMB or TGC were classified as XDR (29).

### Combination antibiotic susceptibility testing

Combination testing was performed according to expert consensus for antimicrobial testing of carbapenem-resistant gram-negative bacteria (30). Briefly, two antibiotic-impregnated paper strips were placed 3 mm–4 mm apart on Mueller-Hinton agar plates pre-inoculated with the test isolate. As antibiotics diffused from the strips, concentration gradients formed inhibition zones. The interactions between drugs were evaluated based on zone enhancement or suppression, classified as synergistic, additive, indifferent, or antagonistic. A total of 140 MDRAB strains were tested, with one independent experimental run performed per strain.

### Mouse infection model construction and MNO + CSL antibiotic regimen validation

Male C57BL/6 mice (6–8 weeks old, weighing 20 ± 2 g) were purchased (Guangdong Zhiyuan Biomedical Technology Co., Ltd.) and adaptively housed in a specific pathogen-free environment for 5 days. They were then randomly divided into four groups, with six mice in each group. The grouping and treatments were as follows: the Control group received only intranasal instillation of 20 μL phosphate-buffered saline (PBS); the antibiotic-only group was administered intraperitoneal injection of CSL (6 mg per mouse per day, once every 12 hours) combined with intragastric administration of MNO (1 mg per mouse per day, once every 12 hours) without bacterial inoculation; the *A. baumannii* group (model group) was intranasally inoculated with 10^8^ (colony-forming units [CFU]) of *A. baumannii* in a 20 μL volume without antibiotic treatment; the *A. baumannii* + MNO + CSL group (treatment group) received the antibiotic intervention according to the above regimen 2 hours after intranasal inoculation of 10^8^ CFU (20 μL) of *A. baumannii*. Forty-eight hours after infection, all mice were euthanized. Lung samples were collected by dissection for bacterial load analysis and determination of the lung wet/dry weight ratio. Concurrently, lung tissue samples were collected for hematoxylin-eosin (H&E) staining pathological analysis, and the cytokine levels in the bronchoalveolar lavage fluid (BALF) were detected.

For histological and pulmonary edema assessment, mouse lung tissues were fixed with 4% paraformaldehyde, embedded in paraffin, and sectioned into 4 μm-thick slices. The sections were stained with H&E following standard procedures, and the histopathological changes were observed under a light microscope (Olympus, Tokyo, Japan). To determine pulmonary edema, the surface moisture of the lung tissue was blotted dry, and its wet weight (W) was measured immediately. The tissue was then dried at 65°C for 48 hours, and its dry weight (D) was measured. The degree of tissue edema was evaluated by calculating the W/D ratio.

BALF samples collected from mice in each group were centrifuged at 1,000 × *g* for 10 minutes at 4°C to separate cells and supernatant. After lysing red blood cells, the remaining cells were resuspended in 1,000 μL PBS for the determination of total cell count using an automatic cell counter. BALF cells were spread on glass slides by the cell smearing method and stained with Wright-Giemsa Stain (Leigen Biotechnology, Beijing, China) for differential cell counting. Meanwhile, the levels of interleukin-1β (IL-1β), interleukin-6 (IL-6), and tumor necrosis factor-α (TNF-α) in the supernatant were detected using enzyme-linked immunosorbent assay kits (Jianglai Biotechnology, Shanghai, China).

### Survival experiments using the *Galleria mellonella* infection model

Bacterial suspensions were adjusted to 0.5 McFarland standard (1.5 × 10⁸ CFU/mL) in 1× PBS, then diluted to 10⁷ and 10⁶ CFU/mL. All seven ST697 isolates were tested. *A. baumannii* ST2 isolate Aba220968 (containing all detected virulence genes) served as a positive control, while PBS was the negative control. The experiments were conducted in two batches, and for each batch, the CFU inoculum was verified prior to the start of the experiments. Specifically, healthy, uniformly sized larvae (free of pigmentation) were selected after incubation at 37°C. Each group of 20 to 30 larvae was injected with 10 μL of bacterial suspension into the last left proleg (Table S24). Each condition was tested in duplicate. Injured larvae were replaced. After injection, larvae were incubated in sterile Petri dishes at 37°C for 72 hours. Survival was assessed at 0, 2, 4, 6, 8, 10, 12, 24, 36, 48, and 72 hours (31). Larval viability was determined by response to gentle prodding with fine-tipped forceps; dead larvae appeared darkened and non-responsive and were removed during each observation.

### DNA extraction and sequencing

Genomic DNA was extracted using the QIAamp DNA Mini Kit (Qiagen, Germany) and quantified with a Qubit 2.0 Fluorometer (Thermo Fisher Scientific, USA) following the manufacturer’s instructions. DNA was fragmented via sonication, and sequencing libraries were prepared using the NEBNext Ultra DNA Library Prep Kit for Illumina (NEB, USA). Libraries were sequenced on the Illumina NovaSeq 6000 platform using 150 bp paired-end reads. For long-read sequencing, DNA from the seven ST697 isolates was used to construct libraries with the LSK-110 kit and sequenced on the PromethION P48 platform (Oxford Nanopore Technologies, UK).

### Bioinformatic analysis

The Illumina raw sequencing reads underwent quality control using FastP (v.0.20.0) (32) with default parameters. The resulting clean reads were assembled into draft genomes using Unicycler (v.0.4.8) (33). Raw Nanopore long reads with a read quality below 10 or a read length less than 8,000 bp were filtered out. For the assembly of ST697, short reads and long reads were combined as inputs for Unicycler (v.0.4.8). All assembled sequences were evaluated using BUSCO (v.5.4.6) (34) with reference to the “bacteria_odb10” database. Species composition identification was performed on the assembled genomic sequences using Kraken2 (v.2.1.2) (35). Coding sequences and non-coding RNA elements were annotated using Prokka (v.1.14.6) (36). MLST analysis was conducted using pubMLST (v2.11) (37). ARGs and VFGs were identified by aligning the protein sequences of annotated CDSs with the reference databases CARD (38) and VFDB (39) using BLASTp (40). Single nucleotide polymorphisms (SNPs) among different genomes were identified using Parsnp (v.2.1.1) (41). The core genome of the isolates was constructed using Roary (42) with the following parameters: “-i 90 -p 40 -o Abpan -cd 95 -v -ap”. The aligned core-gene set sequences were used to construct a maximum likelihood phylogenetic tree using RAxML (43) (https://github.com/amkozlov/raxml-ng) with a bootstrap value of 1,000. The resulting trees were plotted and optimized using iTOL (44) (https://itol.embl.de/).

### Identification of key genomic elements related to antibiotic resistance

Both rule-based and random forest-based approaches were applied to identify key genomic elements associated with antibiotic resistance. Predictive performance was evaluated using seven metrics: accuracy (ACC), precision, recall, specificity, F1-score, mean squared error, and variance of mean squared error. The rule-based method inferred resistance based on the presence of known ARGs. Fisher’s exact test was used to assess the statistical association between the presence of each ARG and resistance to specific antibiotics by comparing resistant and susceptible isolates.

### Statistical analysis

Statistical analysis in this study was performed using R (v.4.1.2). Statistical analyses were performed using Rscript (v.4.1.2). The χ^2^ test (group number ≥10) and Fisher’s exact test (group number <10) were used to compare mortality rates between patients infected with MDRAB and XDRAB. The log-rank test was used to assess differences in survival among groups in the *Galleria mellonella* infection model. A *P*-value <0.05 was considered statistically significant.

## RESULTS

### General characteristics of isolates and patient demographics

A total of 148 clinical MDR isolates, primarily identified as *A. baumannii*, were collected from 26 different wards across three hospital campuses (Table S1). Among these patients, 77.03% had a hospital stay of more than 2 weeks. Of the isolates, 45.95% were collected from ICU wards, and 29.73% from respiratory medicine wards. The majority of the isolates were obtained from respiratory tract specimens (97.30%), including samples obtained via bronchoscopy (45.95%), regular sputum collection (27.70%), deep sputum collection (18.24%), and BALF (5.40%). Furthermore, 95.95% of these infections were classified as hospital-acquired. Among the cases sampled, 24.32% (36/148) of patients ultimately died. Additionally, 53.38% (79/148) of the *A. baumannii* isolates were identified as the primary causative pathogen, with a significantly higher mortality rate in this group compared to the overall cohort (32.91% vs 24.32%, *P* = 0.029).

### Antimicrobial susceptibility profiles

**Fig 1:**
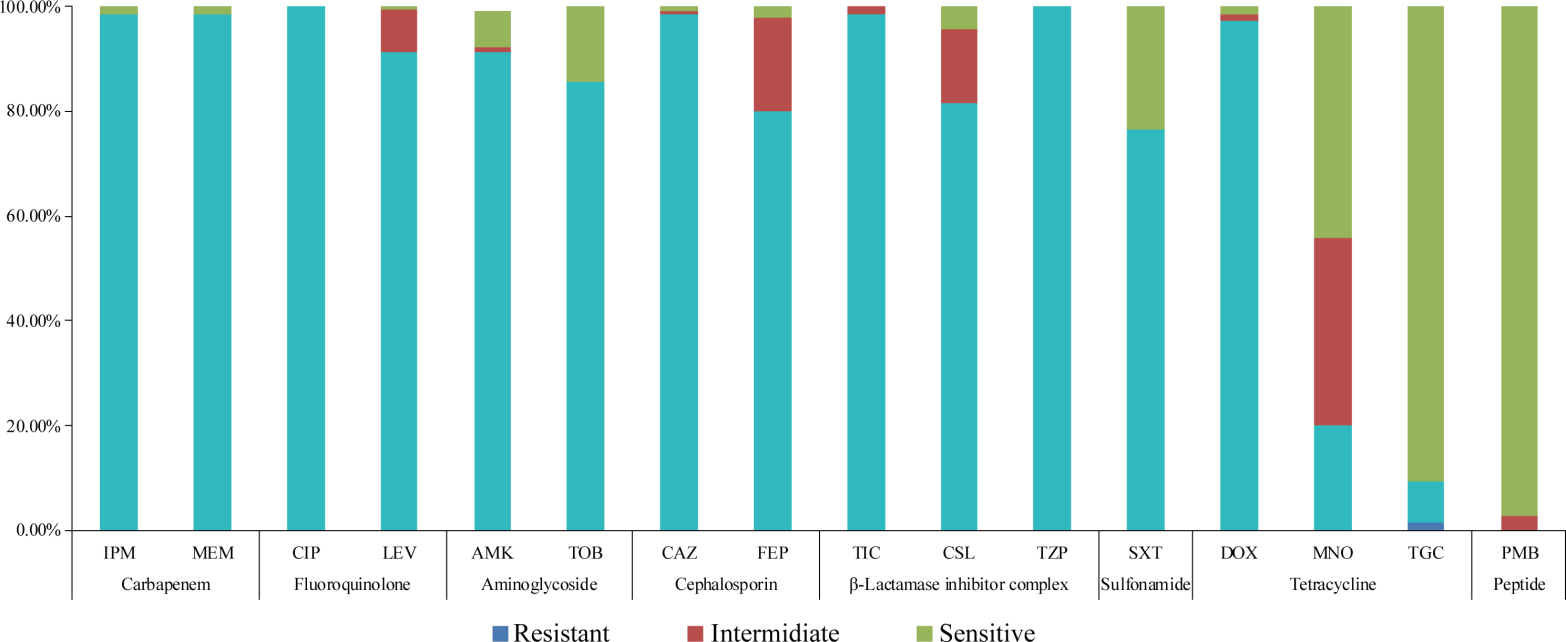
Antibiotic resistance profiles of 140 clinical *A. baumannii* isolates.

A total of 16 antibiotics from eight classes (Tables S2 to S4s) were used for antimicrobial susceptibility testing, with resistance rates ranging from 0% to 100% (Fig. 1). All isolates were found to be MDR, with 45% (63/140) classified as XDR (Table S5). The isolates showed high resistance to fluoroquinolones (100%), β-lactamase inhibitor combinations (100%), cephalosporins (98.57%), carbapenems (98.57%), aminoglycosides (91.43%), and sulfonamides (76.43%). However, they remained relatively susceptible to minocycline (MNO, 44.29%), tigecycline (TGC, 90.65%), and polymyxin B (PMB, 97.14%).

### Antibiotic combination susceptibility testing

**Fig 2:**
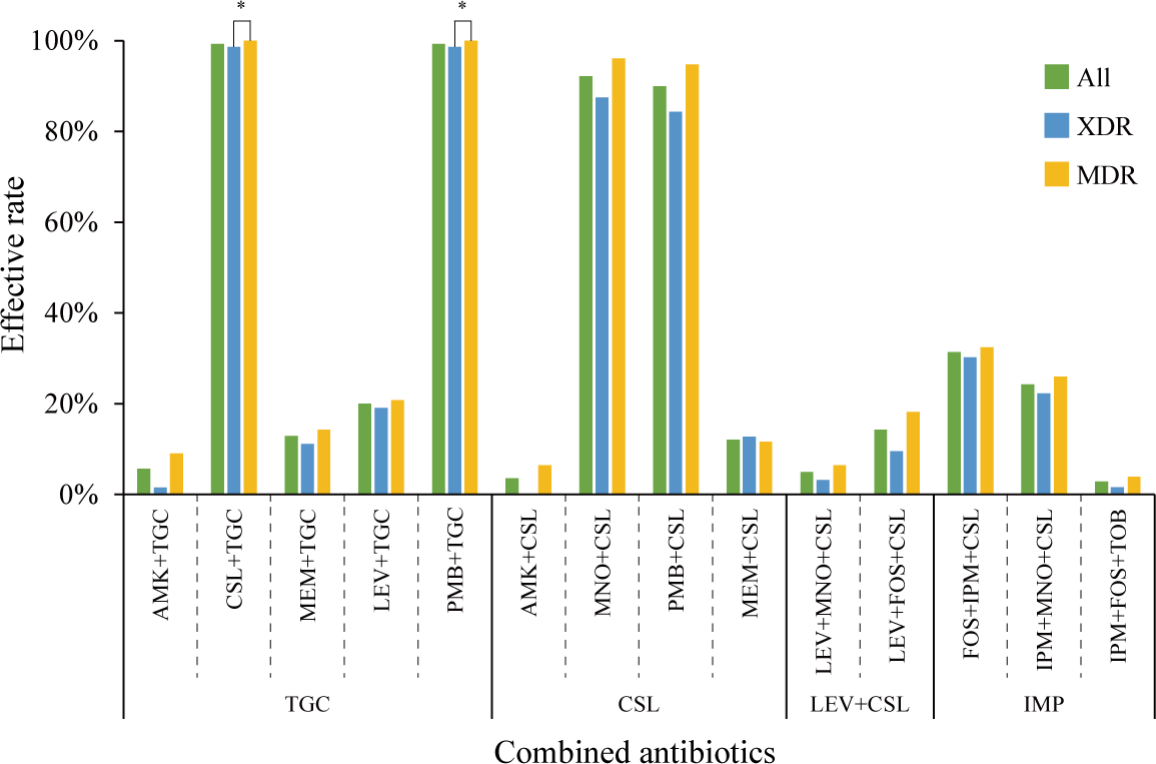
Effectiveness of antibiotic combination therapies across all isolates, MDR isolates, and XDR isolates. “*” indicates corrected *P*-values <0.05. FOS, fosfomycin.

A total of 14 antibiotic combination trials were performed, including nine double combinations and five triple combinations (Table S6). Surprisingly, no strong synergistic effects (<31.43%) were observed in combinations containing carbapenem antibiotics, such as IPM or MEM. Four combinations (45, 46), CSL and TGC (CSL + TGC), PMB and TGC (PMB + TGC), MNO and CSL (MNO + CSL), and PMB and CSL (PMB + CSL), exhibited consistently high activity, with effective rates above 90% (Table S7). Compared to XDR *A. baumannii*, MDR isolates showed significantly higher synergistic efficacy (Fig. 2). Among the four combinations, three include PMB or TGC, which are commonly considered last-line antibiotics for MDR pathogens in clinical practice. The other combination (MNO + CSL), which does not include last-line agents, also demonstrated a strong synergistic effect against both MDR and XDR isolates. This dual combination may offer a promising approach for the clinical treatment of MDR/XDR *A. baumannii*.

### Validation of the combined MNO + CSL regimen in the mouse model

One of the four antibiotic combinations (MNO + CSL) was used to validate its therapeutic efficacy against MDRAB infections. Four experimental groups were established: baseline control group (Control), MNO + CSL-only antibiotic group (MNO + CSL-only), *A. baumannii*-inoculated untreated group (AB-only), and *A. baumannii*-inoculated + MNO + CSL combined antibiotic group (AB + MNO + CSL).

**Fig 3:**
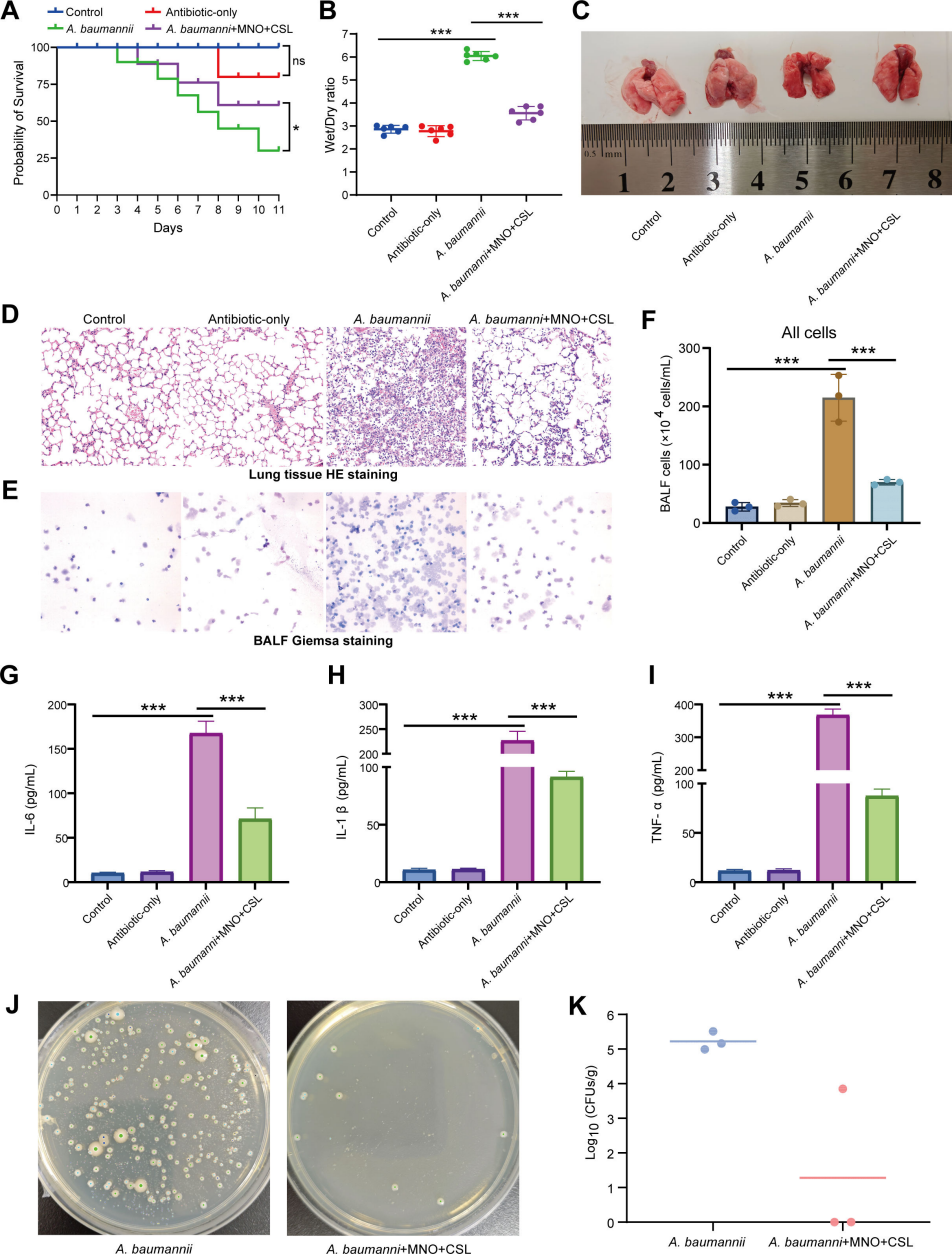
Validation of the combined MNO + CSL regimen in the mouse model. (**A**) Survival curves of four mouse groups (*n* = 6 per group) over 10 days: baseline control group (Control), MNO + CSL-induced antibiotic-only group (MNO + CSL-only), *Acinetobacter baumannii*-inoculated untreated group (AB-only), and *Acinetobacter baumannii-inoculated* + MNO + CSL combined antibiotics group (AB + MNO + CSL); (**B**) lung tissue dry-to-wet weight ratio in mice from different treatment groups; (**C**) representative photographs of lung tissues from mice receiving distinct treatments; (**D**) hematoxylin-eosin (HE)-stained sections of lung tissues from mice in different treatment groups; (**E**) Giemsa staining of BALF from mice with different treatments; (**F**) total cell counts in BALF of mice across treatment groups; (**G–I**) expression levels of IL-1β (**I**), IL-6 (**J**), and TNF-α (**K**) in BALF of mice from each group; (J) bacterial culture results of tissue homogenate smears from *A. baumannii*-infected mice treated with or without intranasal MNO + CSL combined antibiotics; (K) quantitative analysis of *Acinetobacter baumannii* bacterial loads. “*” and “***” indicate statistical significance at *P* < 0.05 and *P* < 0.001, respectively. ns, not significant.

In terms of survival rate, the MNO + CSL-only group and control group exhibited the highest survival rates, with no significant difference between the two. In contrast, the *A. baumannii*-infected group had the lowest survival rate. Compared with the AB-only group, MNO + CSL treatment significantly improved the survival rate of infected mice (Fig. 3A). Analysis of the lung W/D ratio revealed no significant statistical difference between the uninfected control group and the MNO + CSL-only group. Compared with the uninfected control group, the lung W/D ratio was significantly increased in the AB-only group. Notably, after treatment with the MNO + CSL combined antibiotic regimen, the lung W/D ratio of *A. baumannii*-infected mice was significantly reduced but remained slightly higher than that of the uninfected control group (Fig. 3B).

H&E staining of lung tissue showed that compared with the uninfected control group and MNO + CSL-only group, the AB-only group exhibited significantly increased staining density, characterized by massive inflammatory cell infiltration, exudate accumulation in the alveolar lumen, thickened pulmonary interstitium, and disruption of normal alveolar structure (Fig. 3C and D). In contrast, after treatment with the MNO + CSL combined antibiotic regimen, the staining density of lung tissue in AB-only mice was significantly reduced, accompanied by decreased inflammatory cell infiltration and partial restoration of alveolar structure.

Giemsa staining of BALF showed that compared with the uninfected control group and antibiotic-only group, the AB-only group exhibited a significant increase in the number of BALF cells, predominantly neutrophils (accounting for >60% of total cells) with activated morphological features (increased nuclear lobulation and translucent cytoplasm) (Fig. 3E). In contrast, after treatment with the MNO + CSL combined antibiotics, the number of BALF cells in *A. baumannii*-infected mice was significantly reduced, the proportion of neutrophils decreased, and the proportion of macrophages recovered to a level close to the control group with normalized cell morphology. The total cell count (×10⁶/mL) of BALF in the AB-only group was significantly higher than that in the uninfected control group and antibiotic-only group (*P* < 0.01). The total cell count in the MNO + CSL-treated group was significantly lower than that in the AB-only infected group (*P* < 0.01) but slightly higher than that in the uninfected control group (*P* > 0.05) (Fig. 3F). These results demonstrate that the combined antibiotic regimen effectively alleviates MDRAB-induced pulmonary inflammatory cell infiltration, though it has not yet fully returned to the normal physiological state.

Detection of three key inflammatory cytokines (IL-6, IL-1β, and TNF-α) in BALF demonstrated that the expression levels of these cytokines were significantly higher in the AB-only group than in the uninfected control group and antibiotic-only group, indicating a robust pulmonary inflammatory response induced by *A. baumannii* infection (Fig. 3G through I). Importantly, MNO + CSL combined antibiotic treatment significantly reduced the levels of these inflammatory cytokines in *A. baumannii*-infected mice, effectively alleviating pulmonary inflammation (Fig. 3G through I). This finding is highly consistent with the aforementioned results from lung histopathological analysis (HE staining), wet-to-dry weight ratio, and BALF cell assays, further validating the anti-inflammatory efficacy of the treatment at the molecular level.

Additionally, lung homogenate culture assays revealed a significant decrease in *A. baumannii* CFU following MNO + CSL treatment (Fig. 3J and K). This result directly confirms the bacteriostatic/bactericidal activity of the combined antibiotic regimen against MDRAB, providing further evidence for its therapeutic effectiveness.

### Molecular epidemiology based on MLST

All isolates underwent whole-genome sequencing using next-generation sequencing platforms (Table S8). The clean data were assembled into draft genomes, and species identification revealed that 140 isolates were pure *A. baumannii* clones. These genome sequences were of high quality, with an average genome size of 3.98 Mb, a contig N50 of 150 kb, and a BUSCO completeness score of 98.39% (Table S9).

When compared to the publicly available genome data set (*n* = 19,672), a significantly higher proportion of isolates in this study were identified as ST2 (92.86% vs 62.25%, *P* < 0.0001). Additionally, seven isolates were classified as ST697 (5%), and three isolates were identified as ST25, ST109, and one unknown type (Tables S10 and S11). Previous studies have consistently reported a strong association between ST2 and MDR, with ST2 being the predominant molecular type both in China and globally. In contrast, ST25 (1.67%, 331) and ST109 (0.10%, 20) were infrequent in the public data set, primarily representing cases outside of China. Notably, ST697 has not been reported in any previous studies, and only one genome was found in the public data set, which was also isolated from Guangzhou, China. These findings strongly suggest that ST697 may represent a novel molecular type undergoing rapid spread.

### Emergence of early dissemination of a novel sequence type, ST697

To investigate the relationships among isolates, a maximum likelihood phylogenetic tree was generated using the core gene sets of all isolates. The ST2 *A. baumannii* isolates were found to be closely clustered (Table S12), as were the ST697 isolates. A total of only 65 SNPs were identified within ST697 (Aba222462, Aba222137, Aba221002, Aba221468, Aba221262, Aba222537, Aba222489) (Table S13), suggesting that these isolates may belong to closely related clones.

All ST697 cases were identified as hospital-acquired infections, indicating that ST697 has already spread within the hospital. Tracing the infected hosts revealed that ST697 isolates were collected from five wards across two hospital campuses, suggesting possible cross-campus transmission. Based on the timeline, all ST697 isolates were collected within the last 5 months of the sampling period, and the detection rate of ST697 was rising rapidly (Fig. S1). These findings strongly indicate that ST697 is currently in the early stage of emergence with a potential for dissemination.

### ST697 exhibits high similarity to ST2 in VFG and ARG profiles

**Fig 4:**
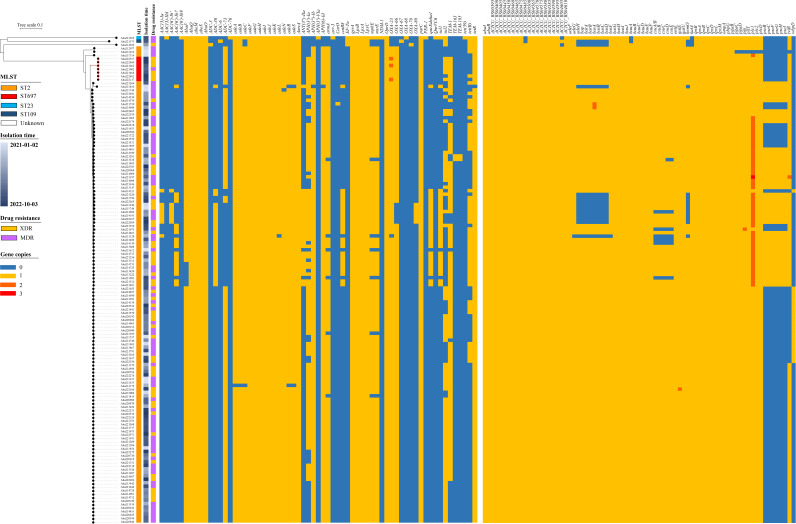
Antibiotic resistance genes and virulence genes in 140 *A. baumannii* isolates. From left to right: phylogenetic tree, isolate names, MLST types, isolation times, resistance classifications, and heatmaps of resistance genes and virulence genes (orange: positive; blue: negative).

Based on the results of AST experiments, ST697 isolates demonstrated a remarkable level of drug resistance, classifying them as XDR, with resistance observed against all antibiotics except for PMB and TGC. Genomic analysis of ST697 revealed the presence of a substantial number of critical resistance genes (Fig. 4; Table S14). Notably, all ST697 isolates were found to harbor two OXA-type β-lactamase genes, namely *bla*_OXA-23_ and *bla*_OXA-66_, which confer strong resistance to carbapenems. Additionally, the TEM-type β-lactamase gene *bla*_TEM-1_, strongly associated with penicillin resistance, was also detected in all ST697 isolates. Furthermore, the sulfonamide-resistant dihydropteroate synthase gene *sul2* was also identified in ST697, conferring resistance to sulfonamide antibiotics.

**Fig 5:**
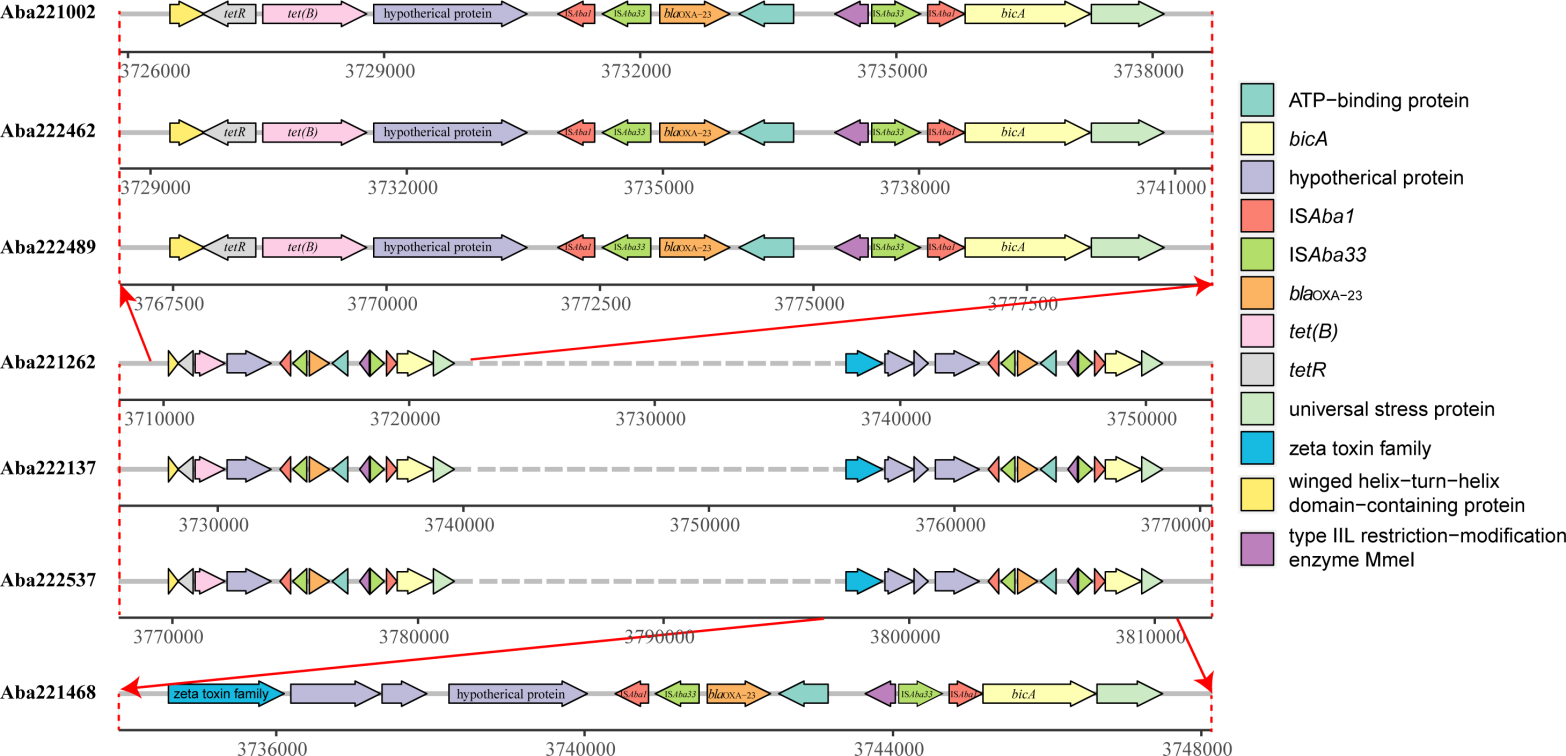
Genomic distribution of *bla*_OXA-23_ genes in ST697 isolates. Arrow directions represent gene orientation (forward/reverse), and colors indicate different genes.

Three ST697 isolates (Aba221162, Aba222137, and Aba222537) were found to harbor two copies of *bla*_OXA-23_ (Fig. 5). Comparative analysis indicated that these copies are located in close proximity within the genome. Furthermore, intact insertion sequences were identified upstream of these genes, suggesting that these transposons may be active and contributed to gene duplication.

ST697 harbors nearly all the virulence factors identified in ST2, except for *pseBCFGHI* and *wbpD* (Table S15). We also compared the pre-treatment clinical test results of patients infected with ST2 and ST697 isolates. Our findings indicated that, except for a higher hemoglobin concentration in the ST2 group, there were no significant differences in other test indicators (Table S16 and S17). These observations suggest that ST2 and ST697 may exhibit similar virulence during infection. Collectively, these findings support the conclusion that ST697 is a highly virulent and extensively drug-resistant molecular type.

### ST697 originated from ST2

The analysis of the phylogenetic tree indicates a close relationship between ST697 and ST2 (Fig. S2). This finding is consistent with a single variation in the genotyping of the MLST loci (Table S10) and is further supported by the minor divergence observed in the core SNP analysis (Table S12 and S13). These findings suggest that ST697 may have evolved from ST2 or shares a common ancestor with it. Furthermore, analysis of the SNP difference matrix shows that the ST2 isolates originate from multiple distinct clones, with several ST2 clones currently undergoing broad dissemination.

**Fig 6:**
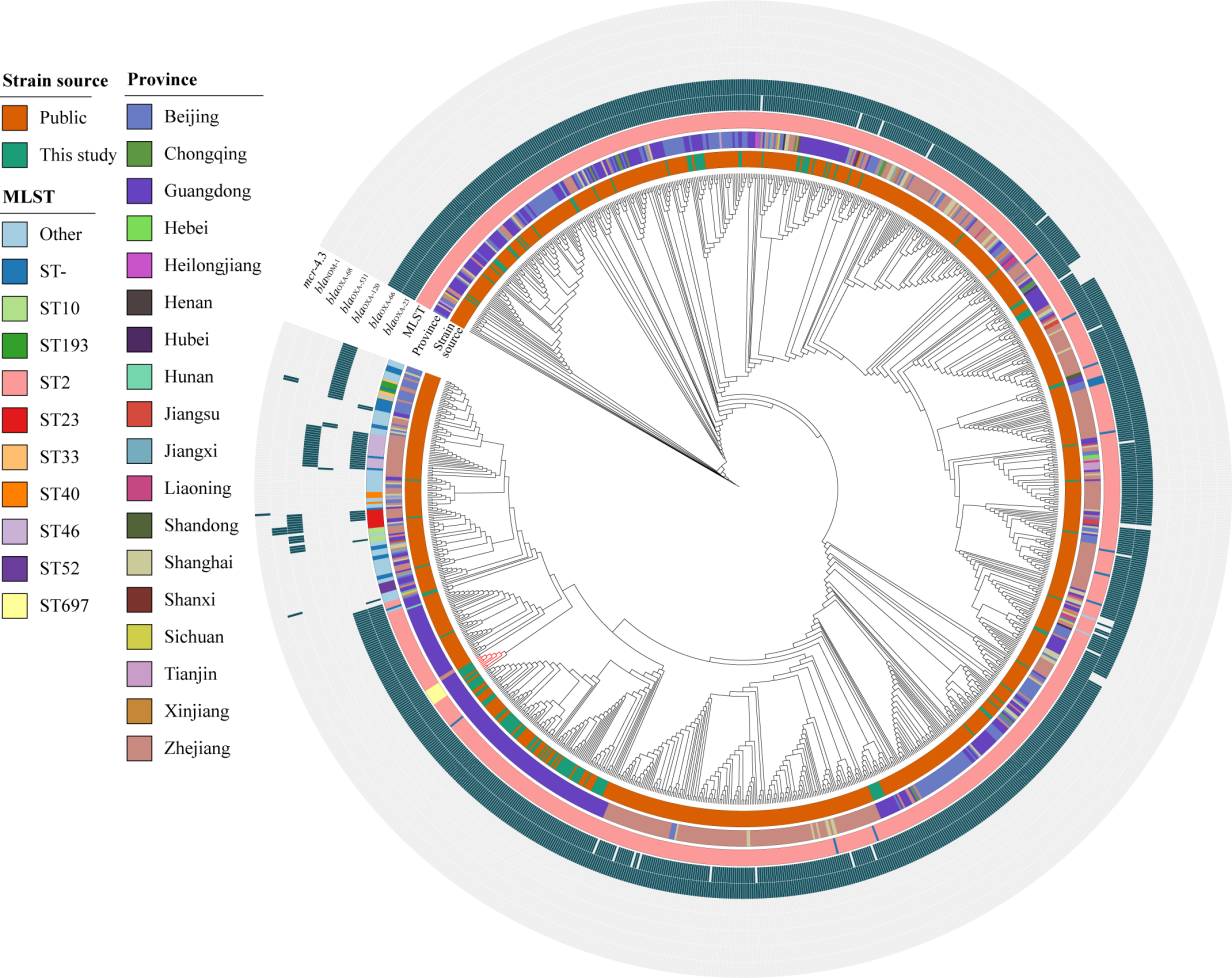
Phylogenetic relationship between ST697 and ST2 isolates. The tree includes all pure isolates sequenced in this study and publicly available *A. baumannii* genomes from hospitals in China. From outermost to innermost rings: presence of seven resistance genes, sample sources, geographic origins (provinces, municipalities, and autonomous regions), and MLST types.

To further investigate the origin of ST697, we constructed a phylogenetic tree using all publicly available *A. baumannii* genomes (*n* = 1,001) from hospitals in China (Fig. 6; Table S18). These genomes were isolated from 33 institutions located in 18 provinces, spanning the years 2013–2023. Despite the inclusion of more reference genomes, all ST697 isolates—including one public genome—clustered together, indicating a close common origin. In this newly generated phylogenetic tree, the ST697 lineage was located within the ST2 lineage, confirming that ST697 originated from ST2.

### Characteristics of ST697 complete genomes

To further investigate the genomic characteristics of ST697, we utilized nanopore long-read sequencing to generate complete genomes for seven ST697 isolates (Table S19). The genome sizes of these isolates were similar, ranging from 4,062,380 to 4,090,570 bp (Table S20). Four plasmids (Plasmid 1–4) were identified in the ST697 genomes, with sizes of 70,426 bp, 8,731 bp, 2,283 bp, and 2,178 bp, respectively (Fig. S3 to S6). However, no known ARGs or VFGs were directly annotated within these plasmids. The number of plasmids varied from two to four among isolates, with Plasmid 2 present in all seven. These plasmids exhibited different prevalence patterns among ST2 isolates: Plasmid 1 (64.61%), Plasmid 2 (90.76%), Plasmid 3 (3.74%), and Plasmid 4 (0%) (Table S21).

Analysis of core genome alignment indicated generally strong collinearity among the seven ST697 isolates, with no major insertions or inversions observed, although minor structural variations were noted. Among these isolates, Aba222489 and Aba222537 showed high similarity, while the remaining five isolates were more closely related to each other (Fig. S7). Gene family analysis revealed that Aba222489 and Aba222537 share 113 unique genes in their core genomes compared to the others (Fig. S8). Additionally, phylogenetic analysis demonstrated consistent relationships with the collinearity results, further supporting the close genetic connection among these seven isolates (Fig. S9). These results suggest that the seven ST697 isolates likely originated from at least two clones, highlighting considerable genomic diversity within ST697.

### Key features associated with antibiotic resistance

To investigate the determinants of different drug-resistant phenotypes, we performed a correlation analysis between genomic components and drug sensitivity using both a rule-based method and a random-forest-based method. The rule-based method successfully identified key ARGs with high accuracy (>95%) and precision (95%) for five antibiotics (Table S22). Specifically, the ARGs *armA* and *aph(3’)-VIa* were associated with resistance to AMK, *tet(B)* and *tet(39)* with resistance to DOX, *bla*_OXA-23_ and *bla*_NDM-1_ with resistance to IPM and MEM, and *sul1* and *sul2* with resistance to SXT. Additionally, the random-forest method successfully identified key features related to resistance for five antibiotics, including AMK, TOB, MNO, SXT, and CSL (Table S23). Consistent results were observed for AMK and SXT, such as the presence of *armA* for AMK resistance and *sul2* for SXT resistance. However, compared to the rule-based method, the random-forest method identified more features. Among these, numerous insertion sequences (ISs) were identified as contributing features, implying that ISs may play an important role in antibiotic resistance.

### Virulence characteristics of ST697

Upon reviewing the clinical outcomes of patients infected with ST697, it was found that 71.4% (5/7) had a fatal clinical outcome, which is significantly higher than the overall reported mortality rate of 24.3%, suggesting that ST697 may exhibit high virulence. To further investigate this hypothesis, wax moth (*Galleria mellonella*) infection experiments were conducted. Using 1× PBS as a negative control and a clinical ST2 isolate (Aba220968) as a positive control, we evaluated the virulence of all ST697 isolates. Aba220968 was selected as the high-virulence reference strain because its genome contains the highest number of identified virulence genes (76/77), and it was isolated from a patient with a fatal clinical outcome.

**Fig 7:**
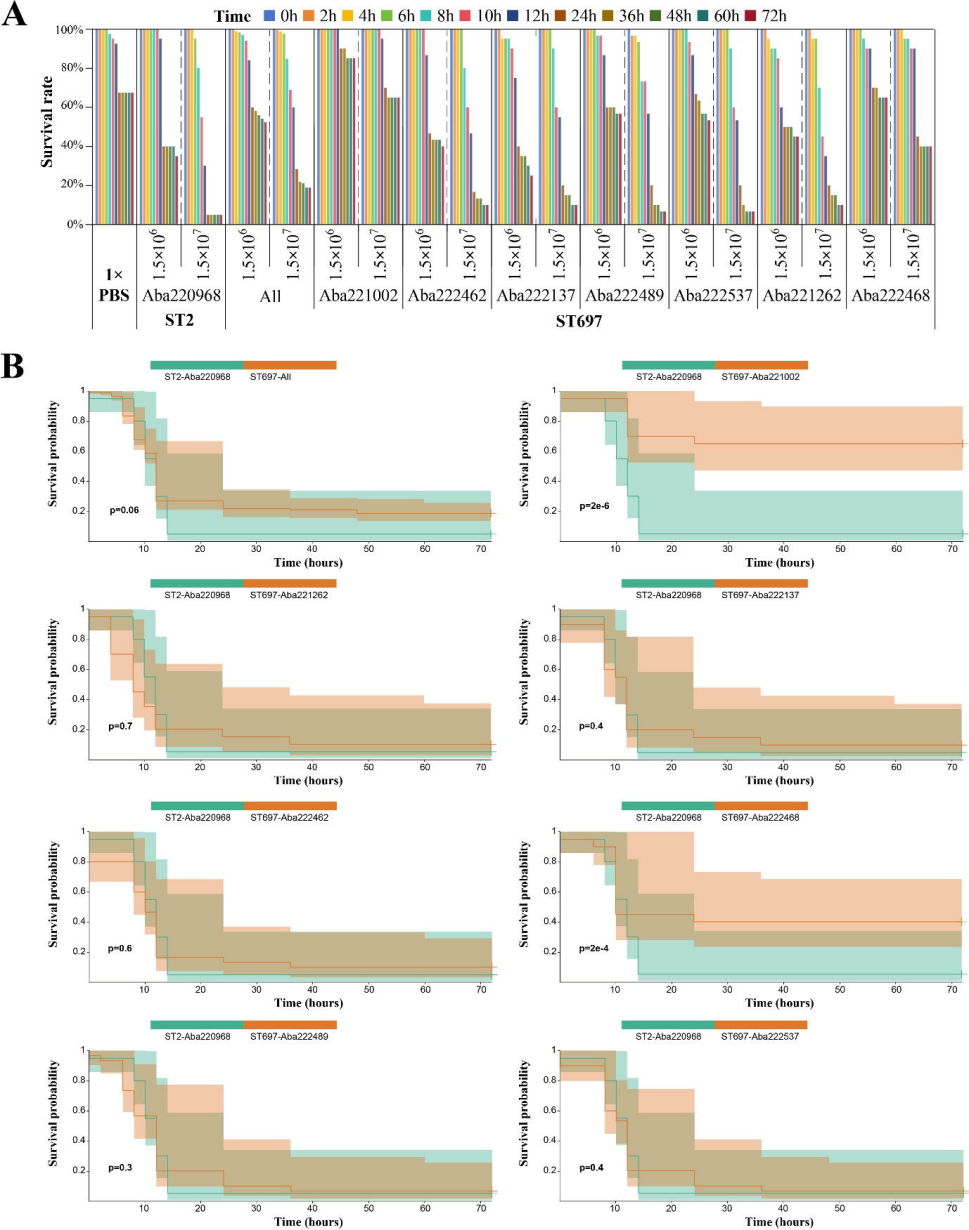
Survival rates in *Galleria mellonella* infection experiments. (**A**) survival rates of larvae at various time points, injection concentrations, and across isolates. 1× PBS served as the negative control; Aba222968 (ST2) served as the highly virulent positive control. “ALL” indicates the combined survival outcome of all ST697 isolates. Bars represent different time points. (**B**) Comparison of survival rates between ST2 and ST697 isolates at an injection concentration of 1.5 × 10⁷/mL. Survival probabilities were compared using the Kaplan-Meier method.

Overall, no significant difference was observed between the survival rates of the ST697 group and the ST2 control strain (Fig. 7A; Table S24). At an injection concentration of 1.5 × 10⁶/mL, there was no significant difference in survival between ST697 and ST2 (*P* = 0.5). When comparing each of the seven ST697 isolates individually to the ST2 control, only one isolate (Aba222137) exhibited a 72 hour survival rate lower than that of ST2 (Fig. S10). At a higher injection concentration of 1.5 × 10⁷/mL, although all isolates exhibited higher survival rates compared to ST2, the overall difference was not statistically significant (*P* = 0.06). When analyzed individually, five of the seven ST697 isolates showed no significant difference in survival compared to ST2, while the remaining two exhibited significantly higher survival rates (Fig. 7B). These results indicate that most ST697 isolates exhibited lethality to *Galleria mellonella* comparable to that of ST2, suggesting similar virulence between ST697 and ST2.

## DISCUSSION

In recent years, *A. baumannii* has emerged as a multi-resistant and often lethal nosocomial pathogen. Although traditionally considered a conditional pathogen, *A. baumannii* exhibits a markedly higher prevalence of antimicrobial resistance compared to other gram-negative bacteria such as *Escherichia coli*, *Klebsiella pneumoniae*, *Pseudomonas aeruginosa*, and *Enterobacter cloacae* (47). This elevated level of drug resistance poses a substantial challenge to effective clinical treatment. In our study, all *A. baumannii* isolates demonstrated multidrug resistance, with an alarming proportion of XDR isolates reaching 42.31%. The isolates exhibited exceptionally high resistance rates to a broad spectrum of antibiotics, with resistance rates exceeding 91%. Fortunately, relative susceptibilities were preserved for PMB and TGC. These findings underscore the urgent need to optimize treatment strategies for MDRAB, as excessive and prolonged use of PMB and TGC may rapidly induce resistance (48).

The implementation of AST has been shown to significantly aid in the selection of effective antibiotic combination therapies in clinical practice (49–51). In this study, we evaluated the efficacy of 14 antibiotic combinations. Our findings revealed that four combinations consistently exhibited synergistic effects *in vitro*, with overall synergistic efficacy rates exceeding 90%. In our data set, all XDR isolates were resistant to at least one of the two antibiotics in each combination. Therefore, the strong synergistic effects observed with these four dual combinations are of considerable clinical significance for the treatment of XDRAB. Notably, for MDRAB, the synergistic efficacy of the four combinations exceeded 95%, while for XDRAB, efficacy remained above 72.6%. Among these combinations, three included PMB or TGC. Although PMB and TGC individually demonstrated favorable therapeutic effects, their combination therapy may reduce the risk of resistance development and help minimize the emergence and spread of pan-resistant strains. Furthermore, our investigation revealed that the combination of MNO and CSL exhibited synergistic effects in 92% of tested isolates. This finding provides a novel therapeutic option for MDR/XDR isolates that does not rely solely on PMB or TGC.

To further validate the efficacy of the aforementioned combination therapy regimens, validation experiments were performed in a mouse model using the MNO + CSL combination. The results demonstrated that, compared to the infection group, several indicators confirmed that MNO + CSL combination therapy significantly ameliorated the condition of mice infected with *A. baumannii*. Specifically, the treatment group exhibited a marked reduction in mortality and a significant decrease in the lung W/D ratio, indicating a notable alleviation of pulmonary edema and a marked alleviation of lung injury (52, 53). Histological analyses of lung tissues and BALF revealed that MNO + CSL treatment effectively reduced inflammatory cell infiltration in the lungs. Additionally, the expression levels of pro-inflammatory cytokines, including IL-6, IL-1β, and TNF-α, were significantly downregulated, further supporting these findings. Culture results of lung tissue homogenates demonstrated that MNO + CSL treatment substantially reduced the *A. baumannii* load in the lungs, directly confirming the anti-infective activity of this combination regimen. In summary, MNO + CSL combination therapy exhibited a significant therapeutic effect in mice infected with MDRAB. Moreover, compared with the normal control group, mice treated with MNO + CSL alone showed no significant differences in mortality, lung W/D ratio, tissue and BALF staining results, or inflammatory cytokine levels, thus confirming the favorable safety profile of this combination therapy regimen.

The emergence of resistance genes is a key driver of antibiotic resistance. These genes may be acquired through various mechanisms, including the production of enzymes that degrade antibiotics, modification of membrane permeability to block drug entry, or activation of efflux systems. Additionally, resistance genes can mediate target site modifications, rendering antibiotics ineffective. Conventional methods for identifying resistance phenotypes rely on bacterial culture, which is time-consuming and often lacks sensitivity, thereby hindering timely resistance profiling. However, with the rapid advancement of metagenomic and targeted metagenomic technologies, identifying key genetic determinants of resistance has become more feasible and impactful (54). By employing rapid pathogen genotyping, resistance phenotypes can be predicted more accurately, thereby reducing time to appropriate therapy (54). This has important clinical implications for improving patient outcomes and limiting resistance development. In this study, we identified key resistance determinants for eight antibiotics, including several ARGs consistent with previous literature, such as *bla*_OXA-23_, *bla*_TEM-1_, and *sul*. Furthermore, beyond ARGs, we found associations between multiple IS elements and resistance. This finding aligns with prior studies (55, 56), suggesting that IS elements may influence the expression of ARGs through interactions with promoters, thus contributing to resistance development. Therefore, when using sequencing techniques to predict drug resistance phenotypes, considering IS elements as additional markers may enhance the accuracy of resistance prediction (57).

WGS is currently regarded as the most accurate method for epidemiological investigations, enabling precise assessment of clonal relationships, detection of outbreaks, and tracking of pathogen evolution (58, 59). In this study, WGS and MLST analysis revealed that the *A. baumannii* isolates were predominantly composed of ST2 and ST697, with ST2 accounting for 92.86% of isolates. This finding is consistent with previous studies identifying ST2 as the most prevalent molecular type (7, 60). Further phylogenetic analysis demonstrated close relationships among ST2 isolates, including several clonal clusters, some associated with hospital outbreaks. This aligns with previous reports (7, 10), confirming the persistent threat of hospital-acquired *A. baumannii* infections.

ST697 represents a novel sequence type that has not been previously reported at the national or global level. From an evolutionary perspective, ST697 appears to have originated from ST2 and shares high similarity in terms of ARG and VFG profiles. Notably, ST697 harbors three carbapenem resistance genes, namely *bla*_OXA-23_, *bla*_OXA66_, and *bla*_TEM-1_, conferring strong resistance to carbapenems. Three ST697 isolates were even found to carry two copies of *bla*_OXA-23_. Additionally, ST697 carries nearly all the VFGs found in ST2, suggesting comparable virulence potential. This was further supported by virulence assays using the *Galleria mellonella* infection model. Alarmingly, a high mortality rate (71.42%) was observed among patients infected with ST697. Although we confirmed that ST697 exhibits comparable virulence to ST2, this high mortality rate may also be influenced by patients’ onset diseases and factors related to the treatment process. Based on temporal and spatial observations, ST697 is currently undergoing rapid dissemination, having been detected in multiple departments across different hospital campuses, indicating a high risk for broader outbreaks. Therefore, it is imperative to strengthen infection control measures, including enhanced disinfection protocols and spatial isolation of ST697-infected patients. Given the high clinical, genomic, and phenotypic similarity between ST697 and ST2, we propose that similar treatment strategies should be applied for managing ST697 infections.

However, the rapid emergence and silent spread of a previously unreported clone in multiple hospital departments underscore the limitations of current diagnostic surveillance and infection control protocols. Since ST697 shares genomic traits with ST2 but has not yet been widely recognized in global databases, it may evade early detection unless routine WGS is implemented. Moreover, its apparent respiratory tropism, high virulence in both clinical and *Galleria mellonella* models, and resistance to frontline antibiotics suggest a potential for ventilator-associated pneumonia outbreaks, particularly in ventilated ICU patients.

In this study, we identified four combination therapy regimens with universal synergistic effects *in vitro* against MDRAB. However, further clinical trials are required to validate their therapeutic efficacy. Of particular interest is the MNO + CSL combination, which showed strong synergy despite not including last-line agents. Its therapeutic efficacy and safety against MDRAB infections were validated in a murine model, thereby offering a less toxic and more sustainable treatment option in resource-limited settings. Additionally, during the identification of key drug resistance determinants, we encountered limitations due to the relatively small sample size and variability in resistance profiles across different antibiotics. Consequently, several important resistance factors may have been missed. Future research should also assess biofilm-related genes and their contribution to persistent respiratory colonization and treatment failure, especially in mechanically ventilated patients. Ultimately, integrating genomic surveillance with clinical outcome data may offer a more predictive framework for guiding therapy and controlling hospital outbreaks.

This study also has limitations. Specifically, only the strip method was used to verify drug synergy, which may lead to insufficient detection stability. In addition, the virulence study of ST697 was solely conducted using the *Galleria mellonella* model; further virulence evaluation using a murine model is required to obtain more comprehensive results.

### Conclusion

In this study, genomic surveillance identified a novel sequence clone, ST697, which appears to be undergoing rapid dissemination within hospital settings. Evolutionarily derived from ST2, ST697 closely mirrors ST2 in both virulence and resistance profiles, including resistance to carbapenems and preserved susceptibility to last-line agents, but may pose a greater clinical risk given its high observed mortality rate. Furthermore, combined AST identified four antibiotic combinations, some not reliant on polymyxin or tigecycline, that exhibited strong synergistic effects against MDRAB/XDRAB. These findings provide critical insights for curbing nosocomial transmission of MDRAB, particularly in high-risk environments such as ICUs, where respiratory infections are prevalent. They also offer practical guidance for tailoring combination therapies and highlight the utility of genomic surveillance as an early warning system for emerging high-risk clones, with potential for regional or global dissemination. These results also underscore the importance of stringent infection control measures and real-time genomic monitoring to contain emerging XDR threats in clinical environments. Continued integration of clinical, genomic, and phenotypic data will be essential for improving outcomes and preventing the spread of novel XDR lineages.

## Data Availability

The raw data and assembled genomes have been uploaded to the National Genomics Data Center (NGDC) at https://ngdc.cncb.ac.cn/ under accession number PRJCA019225. Detailed information about the public data used in this study is provided in the supplementary materials. The assembled ST697 genomes and annotations are shared on figshare (https://doi.org/10.6084/m9.figshare.30579317).

## SUPPLEMENTAL MATERIAL

Supplemental figures spectrum.02293-25-s0001.docx Figures S1 to S10.

Supplemental tables spectrum.02293-25-s0002.xlsx Tables S1 to S24.

ASM does not own the copyrights to Supplemental Material that may be linked to, or accessed through, an article. The authors have granted ASM a non-exclusive, world-wide license to publish the Supplemental Material files. Please contact the corresponding author directly for reuse.
