# Supplementary material for: Emergence of a high-risk multidrug-resistant Acinetobacter baumannii clone ST697 in nosocomial settings
Source: Microbiol Spectr. 2026 Apr 16;14(6):e02293-25. doi: 10.1128/spectrum.02293-25 (PMC13227986; doi:10.1128/spectrum.02293-25)
Supplement: Supplemental figures — Figures S1 to S10. [file spectrum.02293-25-s0001.docx]

**
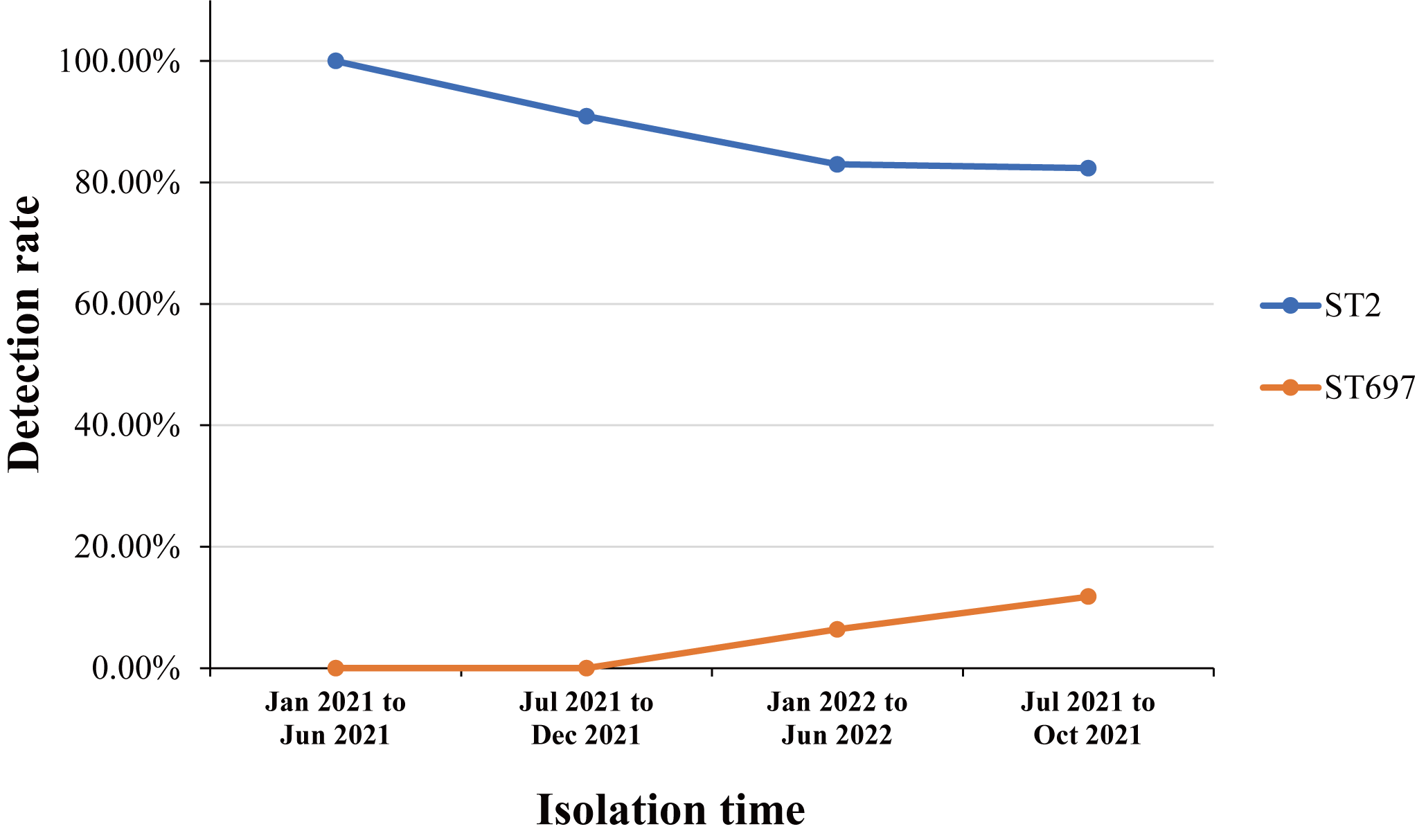
**

**Figure S1. Trends in the detection rates of ST2 and ST697 during the sampling period.**


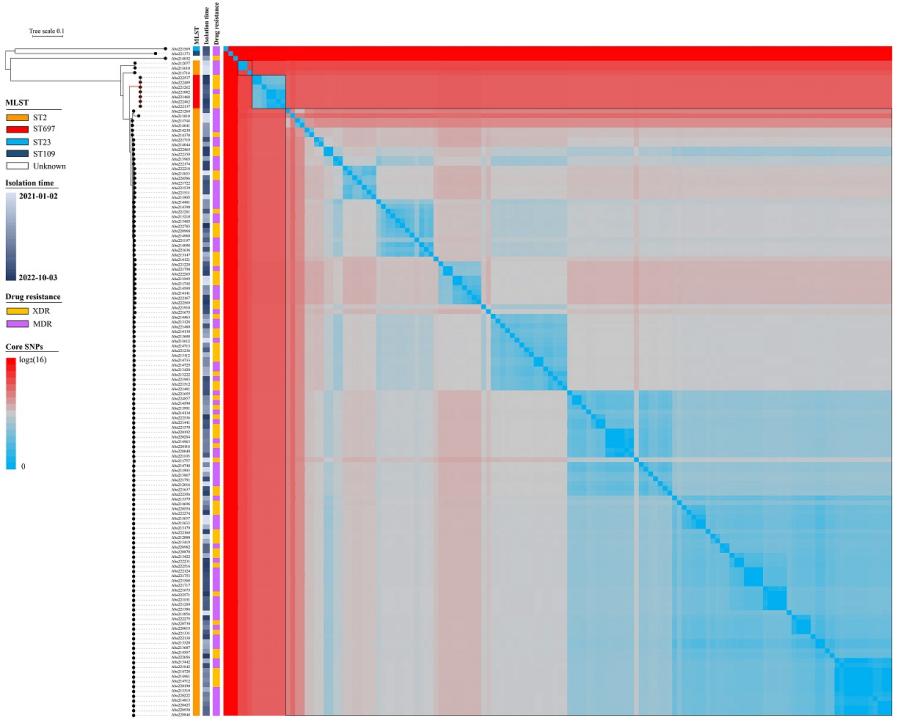


**Figure S2. Phylogenetic distance among** **140 clinical *A. baumannii* isolates.** The tree was constructed using the maximum likelihood method based on core genome SNPs. The accompanying heatmap represents the number of SNP differences between isolates. Blue indicates fewer SNP differences (closer genetic distance), while red indicates more SNP differences. Blue squares in the heatmap suggest very close relationships, implying clonal origin.


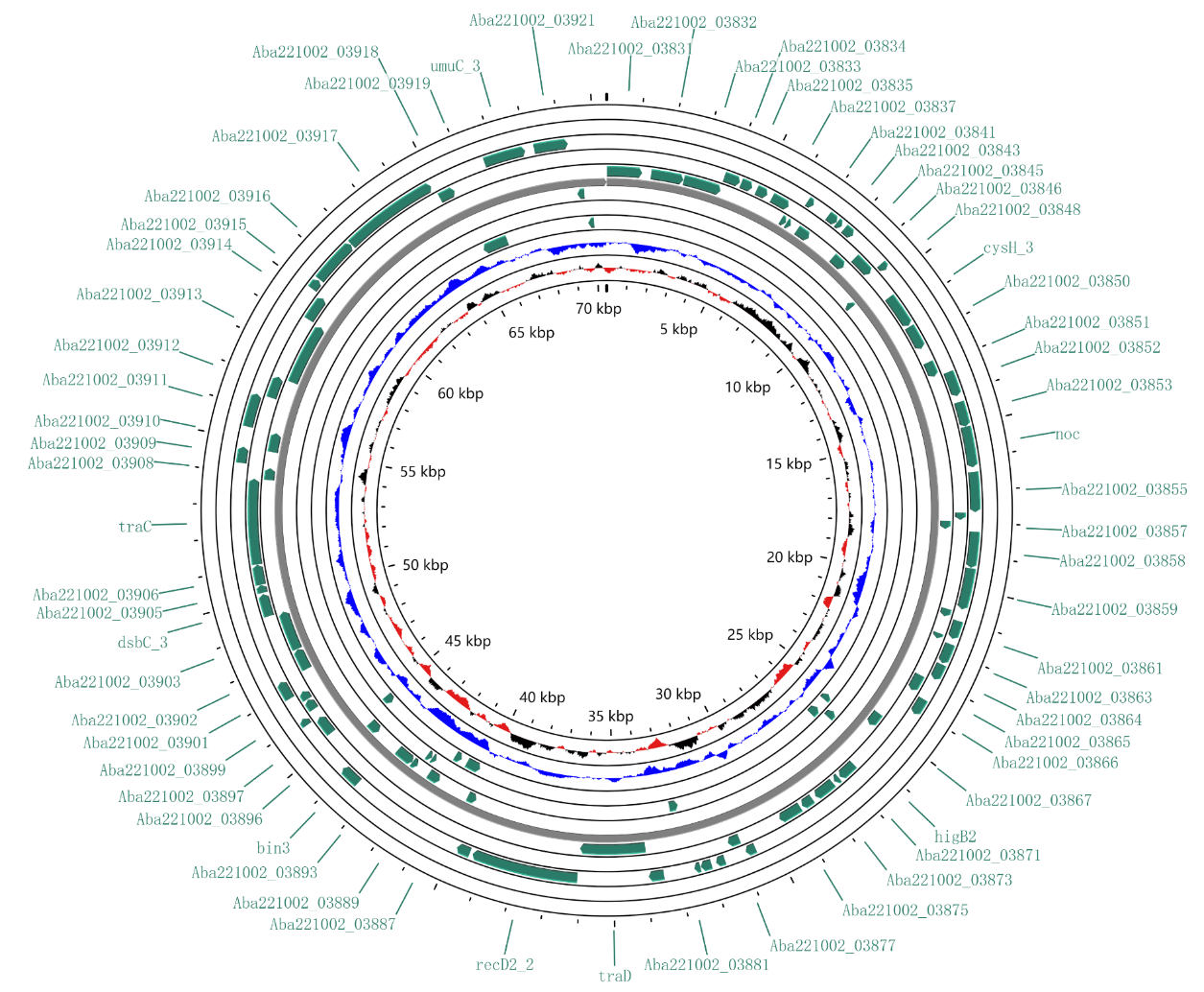

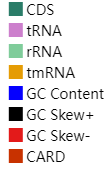


**Plasmid 1**

**70,426 bp**

**Figure S3. Circular plot depicting** **the genomic features of Plasmid 1**. The plasmid genome was annotated using Prokka and visulaized using Proksee. From the inside out, the circular map represents genomic position coordinates, GC skew, GC content, reverse and forward CDS loci, and gene locus names, respectively.


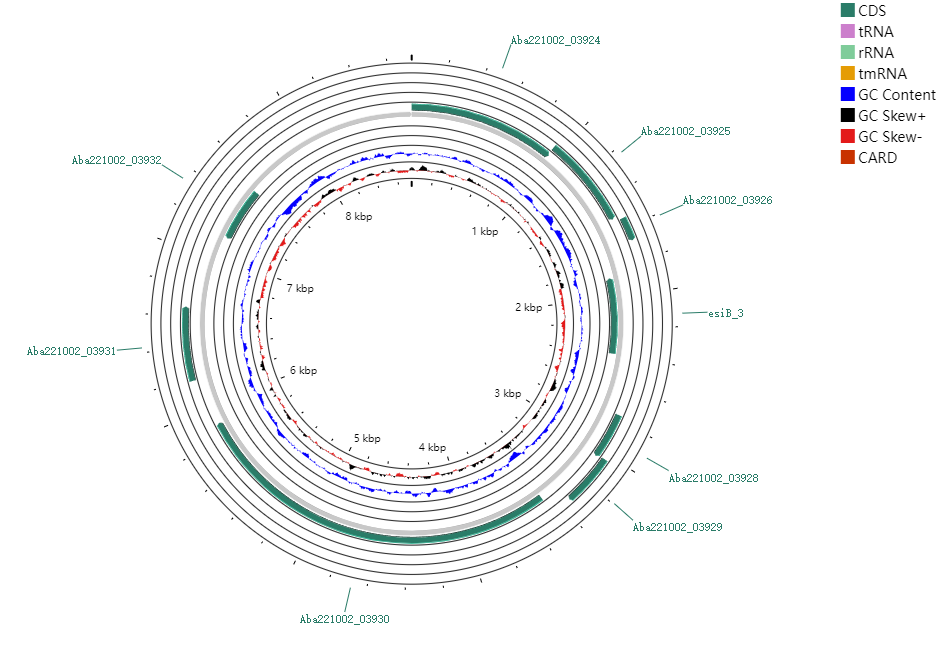


**Plasmid 2**

**8,471 bp**

DNA replication protein

plasmid replication

DNA-binding protein

Hypothetical protein

Secretory immunoglobulin

A-binding protein EsiB

Tetratricopeptide

repeat protein

BrnT family toxin

BrnA antitoxin family protein

TonB-dependent receptor ZnuD

DIP1984 family

protein

hypothetical protein

**Figure S4. Circular plot depicting** **the genomic features of Plasmid 2.** The plasmid genome was annotated using Prokka and visulaized using Proksee. From the inside out, the circular map represents genomic position coordinates, GC skew, GC content, reverse and forward CDS loci, gene locus names and annotated funciton, respectively.


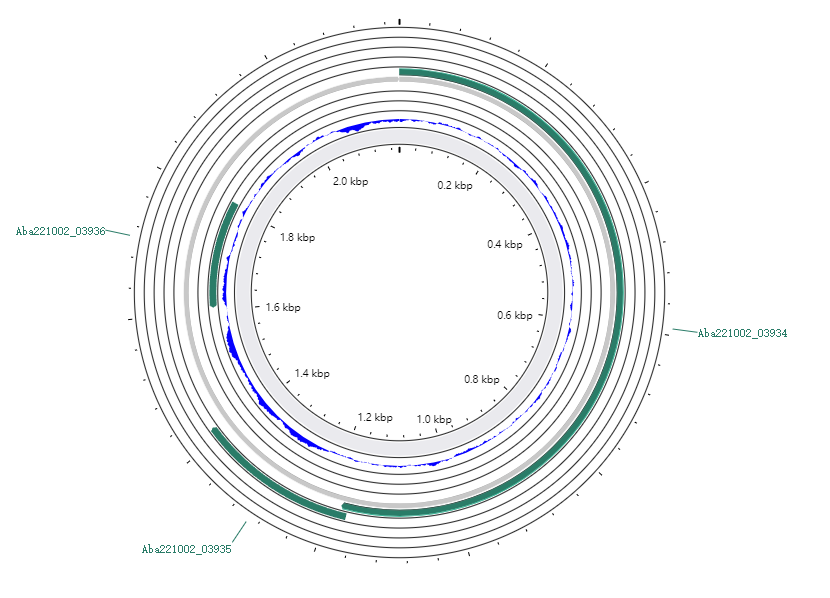


**Plasmid 4**

**2,178 bp**

replication initiation protein

Hypothetical protein

Hypothetical protein

**Figure S6. Circular plot depicting** **the genomic features of Plasmid 4.** The plasmid genome was annotated using Prokka and visulaized using Proksee. From the inside out, the circular map represents genomic position coordinates, GC skew, GC content, reverse and forward CDS loci, gene locus names and annotated funciton, respectively.


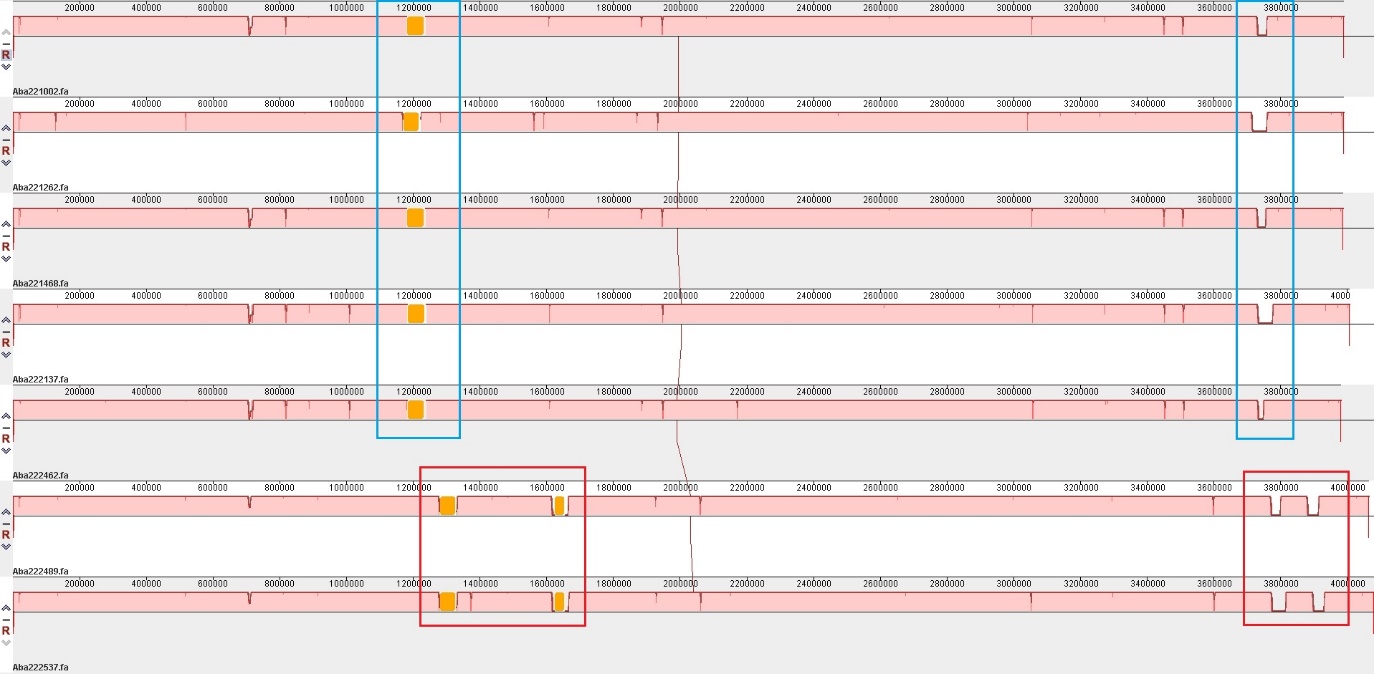


**Figure S7. Collinearity analysis of nuclear genomes of ST697 isolates.**

Genome alignment of seven ST697 strains was performed using Mauve v2.3.1. The red box highlights shared genomic features between Aba222489 and Aba222537. The blue box highlights conserved regions among the other five isolates.


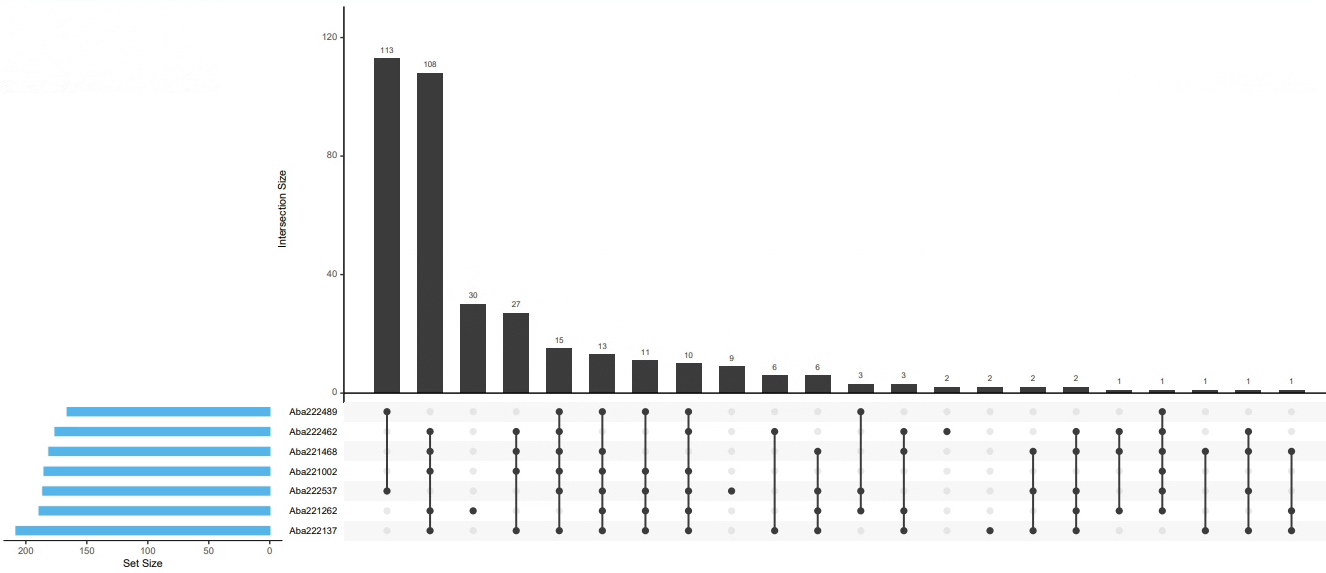


**Figure S8. Upset plot showing the distribution of CDS genes among ST697 isolates**. Black dots indicate genes present in an isolate; grey dots indicate absence. Black bars represent the number of shared genes between groups of isolates. Blue bars on the left indicate the total number of unique genes in each isolate. Aba222489 and Aba222537 share 113 unique core genes not found in the other five isolates. Among the 108 genes shared by the other five strains (second column), 93 differences are attributable to the presence or absence of plasmids. These findings suggest that Aba222489 and Aba222537 are closely related to each other, while the remaining five strains form a separate related group.


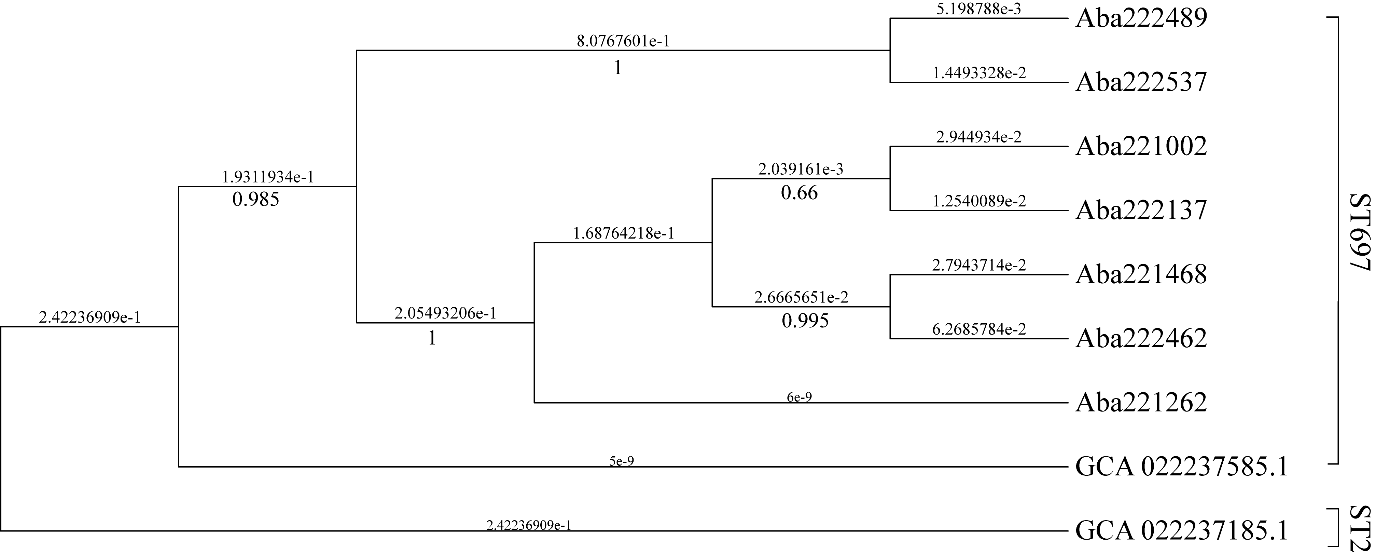


**Figure S9. Phylogenetic relationship among eight ST697 isolates.** A ST2 strain (GCA_022237185.1) was used as the outgroup. Values on the branches represent branch lengths, while those below indicate bootstrap support.


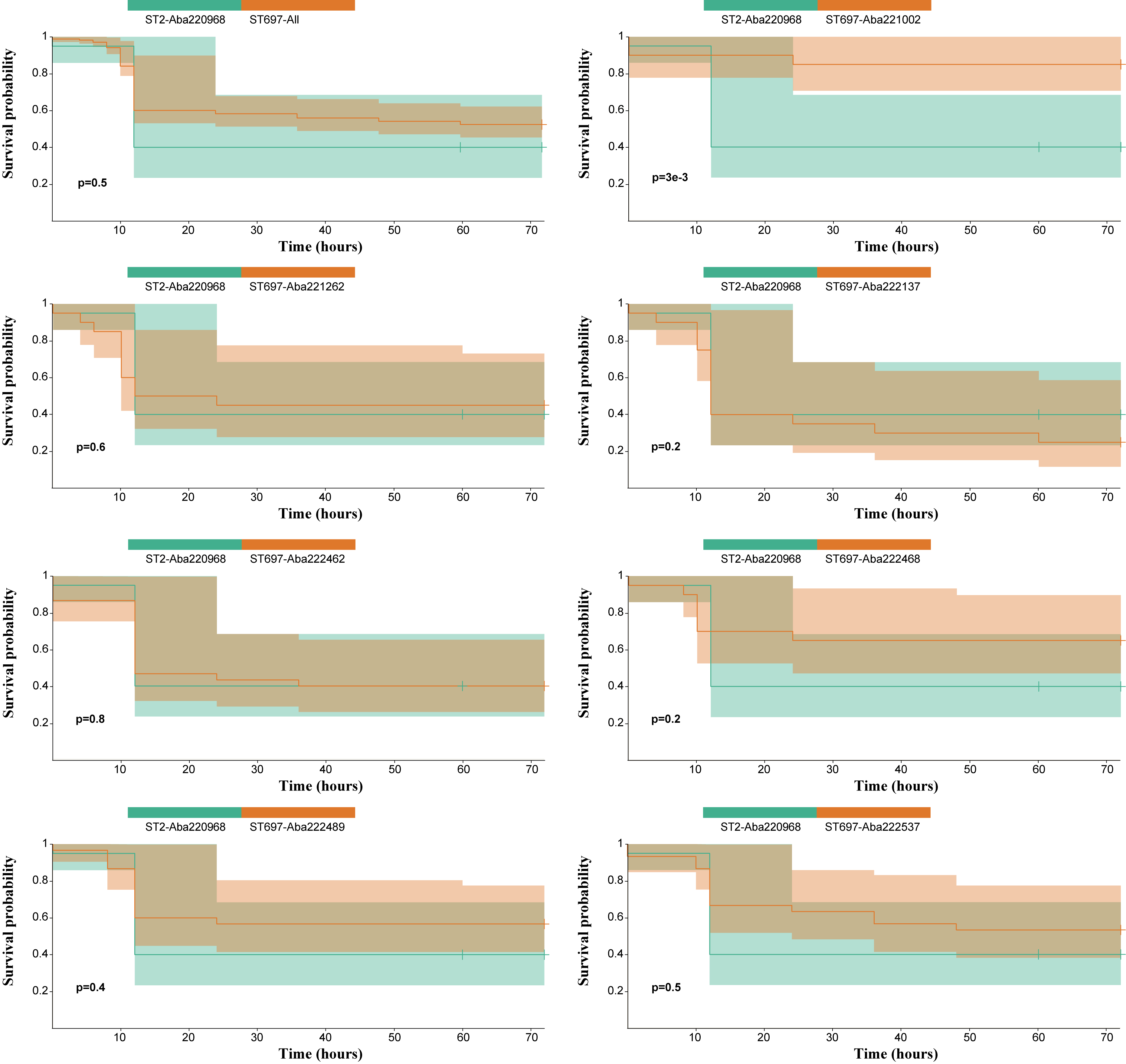


**Figure S10. Survival rate comparison between ST2 and ST697 at an injection concentration of 1.5×10⁶ CFU/mL (72 hours)**. Survival probabilities were compared using the Kaplan–Meier method. The log-rank test was used to assess statistical significance. 1× PBS was set as the negative control and Aba222968 (ST2) was used as the highly virulent positive control.
